# MeCP2: The Genetic Driver of Rett Syndrome Epigenetics

**DOI:** 10.3389/fgene.2021.620859

**Published:** 2021-01-21

**Authors:** Katrina V. Good, John B. Vincent, Juan Ausió

**Affiliations:** ^1^Department of Biochemistry and Microbiology, University of Victoria, Victoria, BC, Canada; ^2^Molecular Neuropsychiatry & Development (MiND) Lab, Centre for Addiction and Mental Health, Campbell Family Mental Health Research Institute, Toronto, ON, Canada; ^3^Institute of Medical Science, University of Toronto, Toronto, ON, Canada; ^4^Department of Psychiatry, University of Toronto, Toronto, ON, Canada

**Keywords:** methyl CpG binding protein 2, Rett syndrome, mutations, protein stability, RNA binding

## Abstract

Mutations in methyl CpG binding protein 2 (MeCP2) are the major cause of Rett syndrome (RTT), a rare neurodevelopmental disorder with a notable period of developmental regression following apparently normal initial development. Such MeCP2 alterations often result in changes to DNA binding and chromatin clustering ability, and in the stability of this protein. Among other functions, MeCP2 binds to methylated genomic DNA, which represents an important epigenetic mark with broad physiological implications, including neuronal development. In this review, we will summarize the genetic foundations behind RTT, and the variable degrees of protein stability exhibited by MeCP2 and its mutated versions. Also, past and emerging relationships that MeCP2 has with mRNA splicing, miRNA processing, and other non-coding RNAs (ncRNA) will be explored, and we suggest that these molecules could be missing links in understanding the epigenetic consequences incurred from genetic ablation of this important chromatin modifier. Importantly, although MeCP2 is highly expressed in the brain, where it has been most extensively studied, the role of this protein and its alterations in other tissues cannot be ignored and will also be discussed. Finally, the additional complexity to RTT pathology introduced by structural and functional implications of the two MeCP2 isoforms (MeCP2-E1 and MeCP2-E2) will be described. Epigenetic therapeutics are gaining clinical popularity, yet treatment for Rett syndrome is more complicated than would be anticipated for a purely epigenetic disorder, which should be taken into account in future clinical contexts.

## Introduction

The term epigenetics has gained much popularity and has gathered the attention of many researchers in recent years. Yet, the term has, at times, been loosely used and quite often in an ambiguous way (Greally, 2018).

In the right context, epi (beyond)-genetics is defined as gene expression alterations resulting from a change in the DNA/chromatin structure which does not involve a change in the underlying DNA nucleotide sequence (i.e., mutations). At the molecular level, this can be elicited by chemical post-replication/post-translational “tags” that mark DNA (Bird, 1993; Greenberg and Bourc’his, 2019), histones (Bannister and Kouzarides, 2011) (the main protein component of chromatin) or other chromosomal and non-chromosomal proteins. These “tags” (for instance DNA methylation (Greenberg and Bourc’his, 2019) or protein modifications) are written and erased through the action of different enzymes (writers and erasers) and read by transcriptional regulatory cofactors (readers) (Marmorstein and Zhou, 2014; Seto and Yoshida, 2014). Such is the case for the methyl CpG binding protein 2 (MeCP2), a DNA methylation reader protein. This protein, initially thought to have repressor activity (Nan et al., 1997), is now recognized to have both transcriptionally repressive and activating functions through its interaction with different cofactors (Yasui et al., 2007; Chahrour et al., 2008). The protein is able to recognize (or read) DNA and histone methylation marks (Lewis et al., 1992; Thambirajah et al., 2012; Lee et al., 2020) and, hence, it acts as a methylation-dependent transcriptional modulator within the context of chromatin (Li et al., 2020). DNA methylation dysregulation is one of the hallmarks of diseases such as cancer (Baylin and Jones, 2016), and MeCP2 mutations can alter the reading of this mark, as in RTT (Kriaucionis and Bird, 2003), where it can impact the normal activity of cells. Interestingly, MeCP2 has been recognized as a *bona fide* oncogene and has been involved in many cancers (Neupane et al., 2015). In any such instance, these diseases often have a genetic origin with downstream epigenetic effects (Yoon et al., 2020).

**FIGURE 1:**
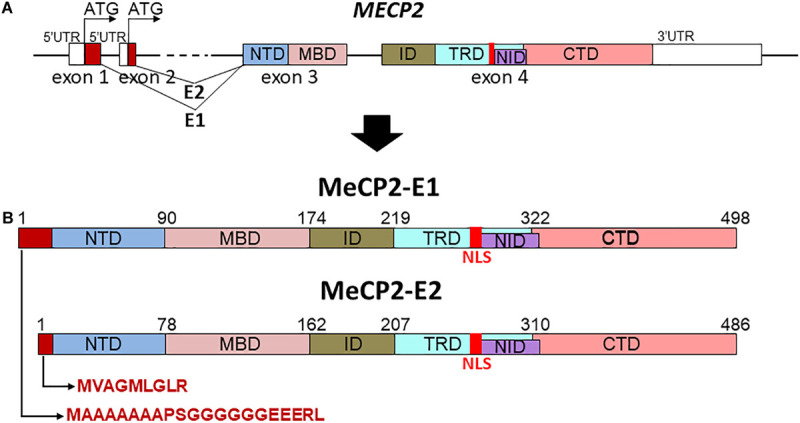
Schematic representation of *MECP2* gene organization **(A)** and different protein domains of the MeCP2-E1 and MeCP2-E2 isoforms **(B)**. NTD, N-terminal; MBD, methyl binding; ID, intervening; TRD, transcription repression; NID, NCoR interaction; CTD, C-terminal domains; NLS, nuclear localization signal.

From structural and functional perspectives, MeCP2 is a good example of an intrinsically disordered protein (IDP) (Dunker et al., 2001; Hite et al., 2012), given its relatively low contents of secondary and tertiary structure organization in solution. Despite this, the protein can be divided into several well-defined structural/functional domains (Ghosh et al., 2010): NTD; N-terminal; MBD, methyl binding; ID, intervening; TRD, transcription repression; NID, NCoR interaction; and CTD, C-terminal; domains (see Figure 1). The TRD includes the nuclear localization sequence (NLS) [amino acids 253–271 in MeCP2-E2 nomenclature (Figure 1B)]. Genetic mutations in the coding region of the X-chromosome-linked *MECP2* gene alter the ability with which its encoded protein MeCP2 binds to DNA within the context of chromatin. In particular, those mutations affecting the MBD of the protein (Figure 1) which affect the stability (Kucukkal et al., 2015) and affinity (Yang et al., 2016) of its DNA binding and which represents an important aspect of this review.

Besides its ability to bind methylated DNA and histones, MeCP2 was earlier recognized (Jeffery and Nakielny, 2004) and more recently confirmed to be (Castello et al., 2016) an RNA binding protein. This less studied facet of MeCP2 will be described next. Following this, we will focus on an equally less understood role of this protein, namely its function in tissues other than those within the brain, and will finally conclude this review with the controversial potential physiological relevance of the two isoforms, MeCP2-E1 and MeCP2-E2 (Figure 1), in RTT (Liyanage and Rastegar, 2014; Martínez de Paz et al., 2019). Because the MeCP2-E2 isoform was the first to be discovered (Lewis et al., 1992), the mutations observed in RTT originally referred to this isoform. Therefore, the amino acid numbers (mutations) referring to the protein sequence of MeCP2 used in the following sections will be those of this isoform (unless otherwise indicated).

## Subsections

### Genetic Origin of Rett Syndrome

Most of the nucleotide transition mutations in Rett syndrome are the C > T type that take place at CpG hotspots (Wan et al., 1999), and likely reflect variable site methylation in the male germline (Cheadle et al., 2000). Hence, all the sporadic mutations, which represent more than 99% of the individuals affected by this syndrome (Chahrour and Zoghbi, 2007), and which involve *de novo* mutations (Comings, 1986) of the *MECP2* gene, are of paternal origin (Trappe et al., 2001). This paternal origin may be explained by a combination of the elevated levels of methylation and mitotic divisions in the male germline (Driscoll and Migeon, 1990; Shahbazian and Zoghbi, 2002). As in the case of other C > T transition mutations, this is likely to involve methyl cytosine oxidative deamination of abnormally methylated cytosines (Tomatsu et al., 2004).

Rett syndrome is almost exclusively a disease that affects girls (XX), yet is not a disease with epigenetic inheritance, such as Prader-Willi syndrome and Angelman syndrome, where the clinical outcome depends on whether a mutation is transmitted from a paternal or maternal chromosome, and RTT mutations are not epigenetic mutations (epimutations) *per se*. Rather, RTT mutations have epigenetic consequences, as MeCP2 is considered to be a reader of epigenetic signals. Although considered a disease affecting girls, this is not completely exclusive. The vast majority of mutations that lead to RTT occur *de novo* in paternal germline cells (Cheadle et al., 2000), and these can only be transmitted to female offspring and never to males. *De novo MECP2* mutations can occasionally be transmitted from mothers, or inherited from mothers who either have mild cognitive impairment or are asymptomatic, due to skewed X-inactivation favoring expression from their wild-type allele.

Rett syndrome clinical features include regression of motor and communicative skills after 6–18 months of apparently normal development [the reader is referred to Einspieler and Marchik (2019) and Banerjee et al. (2019)] for comprehensive descriptions and review of typical and atypical RTT clinical features. While females born with a *de novo* (from mother’s or father’s germ cells) or inherited (from a carrier mother) *MECP2* RTT mutation may have a wide spectrum of severity, males with the same *MECP2* mutations typically have much severer consequences, with a more rapid progression of symptoms and lower average age of death (Neul et al., 2019). However, these may be ameliorated in the presence of an additional X chromosome (Klinefelter’s syndrome), or where the mutation is a somatic mosaic rather than germline. Also, there are reports of males with *MECP2* mutations that are not known pathogenic RTT mutations who are affected, but not with classical RTT (Neul et al., 2019), and some where the clinical presentation includes psychiatric disorders such as schizophrenia (Cohen et al., 2002; Villard, 2007; McCarthy et al., 2014; Curie et al., 2017; Sheikh et al., 2018), bipolar disorder (Sheikh et al., 2016), and Asperger’s (Curie et al., 2017).

### MeCP2 Mutations and the High Complexity of MeCP2 Stability

**FIGURE 2:**
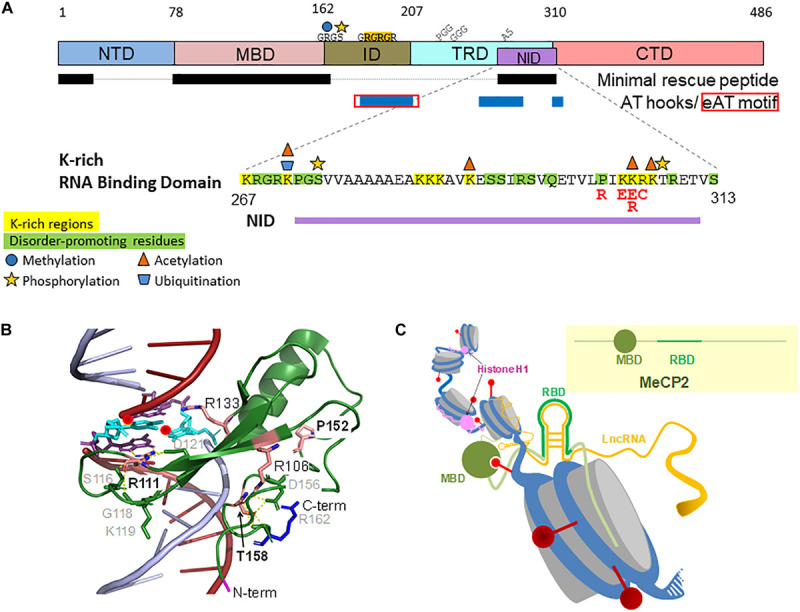
Placement and amino acid sequence and PTMs of the RNA Binding Domain (RBD) within the MeCP2-E2 domains. The main missense RTT mutations are in red **(A)**. Detail of the DNA-bound MBD three dimensional structure (Ho et al., 2008) (Protein Data Bank PDB ID 5BT2) (Berman et al., 2000) **(B)**. Cartoon representation of RNA-bound MeCP2 within a chromatin context **(C)**. Red spheres, DNA methylation; Gray cylinders, nucleosomes.

Rett syndrome can arise from a number of missense, nonsense, frame shift, splice site, and start codon mutations as well as larger deletions that can lead to a range of phenotypes with varying degrees of severity (Chahrour and Zoghbi, 2007). From the protein structural point of view (Figure 1B), MeCP2 mutations can be grouped into three main broad categories. The first corresponds to mutations that affect the NTD, a second, and corresponding to a very significant group of RTT phenotypes, are those that affect the MBD, and a third, those affecting the rest of the molecule. This classification is not arbitrary as the NTD has been shown to modulate the ability of MeCP2 (through the MBD) to interact with DNA (Martínez de Paz et al., 2019) as well as to influence the turn-over rate of the protein (Sheikh et al., 2017; Martínez de Paz et al., 2019), and hence mutations within this region can affect these parameters. With the MBD being the only structurally ordered portion of MeCP2, mutations within the MBD can affect the tertiary structure (folding) (Figure 2B; Kucukkal et al., 2015) of this region and hence its binding affinity (Yang et al., 2016). Many of the remaining mutations are located in the C-terminal domain (Moncla et al., 2002; Bebbington et al., 2010) and can affect the interactions of MeCP2 with many of its diverse interaction partners (Lyst et al., 2013), including the chromatin (Nikitina et al., 2007a) itself and RNA (see following section).

The first attempts to study the functional correlation between RTT MeCP2 mutations and the impairment to DNA methyl binding and transcriptional regulation activities were carried out in the late Alan Wolffe’s lab (Yusufzai and Wolffe, 2000) soon after the discovery of their involvement in this disease (Amir et al., 1999). These initial results were subsequently followed by a detailed characterization of the binding affinity alterations caused by several missense mutations within MBD (Ballestar et al., 2000) and set the framework for the type of work which will be described in the following sections.

#### MeCP2 N-Terminal Mutations

Although not as frequent as the mutations affecting the MBD or TRD at their C-termini (Shah and Bird, 2017; Spiga et al., 2019), several NTD mutations have been described to date and are more often than not associated with a typical RTT phenotype (Saunders et al., 2009). However, as with mutations elsewhere in MeCP2, other factors, skewing of X-inactivation, for example, play a role. Without the ability to compare clinical severity across a larger number of RTT girls with the same N-terminal mutation, and without identification of males with these mutations, it is difficult to draw any firm conclusions. However, a study of clinical severity, albeit with only five cases with N-terminal mutations, of which four displayed typical RTT, suggests on average lower clinical severity in comparison to the common nonsense mutations and missense mutations such as T158M (Cuddapah et al., 2014). It is important to note that the two isoforms differ at the N-terminal sequences, with the MeCP2-E2 N-terminus encoded by exon 2, and the slightly longer MeCP2-E1 N-terminus coming from exon 1 (Figure 1). It is also worth emphasizing that, until recently, no unequivocal RTT mutations have been reported within exon 2 (or indeed for any clinical entity). Very recently, however, there is a report of a NM_004992:c.7G > C; p.Ala3Pro Rett mutation in exon 2 (Wen et al., 2020). Given its rarity, it will be important to assess the molecular effects of this variant to confirm its true pathogenicity.

The MeCP2-E1 N-terminus contains polyGGC and polyGGA stretches that encode stretches of alanine and glycine residues, respectively. In-frame insertions and deletions within the polyalanine and polyglycine regions of exon 1 (Figure 1B) have been identified. Although initially they were suggested to be a relatively frequent cause of intellectual disability or developmental delay (Harvey et al., 2007), with the current availability of exome sequence data from large control populations^1^ it is likely that these in-frame indels are unrelated to disease.

Although genuine disease-causing mutations within exon 1 would appear to affect the MeCP2-E1 isoform exclusively, this is not necessarily the case. The first mutation reported in exon 1, an 11 bp frameshifting deletion, was described when the MeCP2-E1 isoform itself was first reported (Mnatzakanian et al., 2004). Since then, the same mutations have been reported in multiple studies (Saunders et al., 2009), and in one study it was shown that, while there was no disruption of transcription of the MeCP2-E2 mRNA, there was interference with, and reduction of translation of MeCP2-E2 protein (Saxena et al., 2006), leading to the possibility that mutations within the MeCP2-E1 N-terminus affect both major isoforms.

However, subsequently, several classic Rett patients were identified with mutations affecting the start codon in exon 1 (Gauthier et al., 2005; Saunders et al., 2009). Levels of mRNA for MeCP2-E1 and E2 were unaffected, and peripheral blood lymphocytes were still positive for MeCP2 antibodies, and thus presumably only able to generate the MeCP2-E2 protein (Gianakopoulos et al., 2012). Additionally, a genuine RTT missense mutation (A2V) within the same N-terminal region of MeCP2-E1 was reported in two patients (Fichou et al., 2009; Saunders et al., 2009). The mutation resulted in an RTT phenotype characterized by severe epilepsy, cognitive impairment and developmental delay, in addition to microcephaly and no language in one patient (Fichou et al., 2009), and is described as classic Rett syndrome in the other (Saunders et al., 2009). Importantly, neither the transcriptional nor the translational properties of MeCP2-E2 were affected as observed in fibroblasts or lymphocytes obtained from the patients (Fichou et al., 2009; Gianakopoulos et al., 2012). An apparent synonymous or silent mutation in exon 1, p.Gly16Gly, that was shown to trigger usage of a cryptic splice donor resulting in a frameshift and premature truncation for the MeCP2-E1 isoform but with no predicted effect on the MeCP2-E2 isoform has also been documented (Sheikh et al., 2013). A study on the cellular and molecular effects caused by the A2V mutation showed that, while it neither impacted the localization of the MeCP2-E1 isoform nor its co-localization with chromatin, it affected the N-terminal co- and post-translational modifications that regulate the physiological turnover of the protein. Complete N-methionine excision (NME) and evidence of excision of multiple alanine residues from the N-terminal poly-alanine stretch of wild type (WT) MeCP2-E1 was observed, whereas the A2V mutant exhibited only partial NME of either methionine or valine and reduced N-acetylation (NA). This resulted in different *in vitro* protein degradation rates between the WT and the mutant. Indeed, a higher proteasomal degradation activity was observed for MeCP2-E1-A2V compared with that of WT MeCP2-E1 (Sheikh et al., 2017). Hence, the etiopathology of this mutation is likely due to a reduced bio-availability of MeCP2 resulting from the defective co-post-translational N-terminal modifications that lead to a faster degradation of the A2V mutant (Sheikh et al., 2017).

Apart from A2V, there are no other published reports of exon 1 missense mutations. In fact, there are remarkably few examples of missense mutations within the N-terminal region leading up to the MBD. One of these few rarities is A59P. The A59P mutation was described in three Tunisian RTT patients with variable scores of clinical severity (Kharrat et al., 2015). Despite the intrinsically disordered organization of the MeCP2 NTD, such an amino acid change was predicted to have an important structural effect on the overall conformation of the protein backbone. However, the structural role and protein stability properties affected remain to be determined. This also applies to all the other NTD mutations described above, with the exception of A2V, and hints to the complexity of the molecular mechanisms probably involved.

#### MeCP2 Missense Mutations

Missense mutations represent the most abundant mutations in RTT [over 70% (Spiga et al., 2019)] and mainly affect the MBD (residues 78 to 162, Figure 1B) where they make up to approximately 45% of the cases (Ghosh et al., 2008), underscoring the primary role of the MBD in the function of this protein. This domain corresponds to the main structured part within this intrinsically disordered protein (Dunker et al., 2001; Ausió et al., 2014), and is the only region that has been amenable to crystallization (Ho et al., 2008). The MBD crystal structure has provided an excellent resource for the analysis of the structural alterations caused by mutations within this region.

**FIGURE 3:**
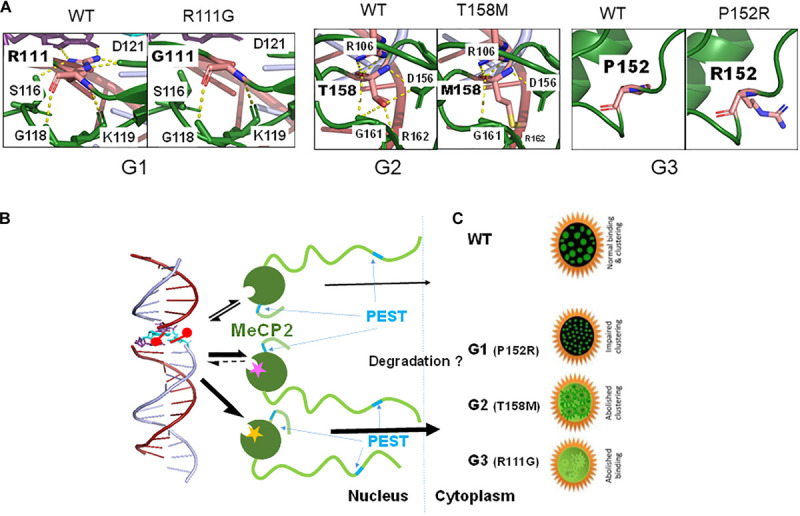
Effect of a few MBD missense mutations [groups G1 to G3 (Yang et al., 2016)] on MeCP2 structure (stability) **(A)**, binding affinity (magenta star: G1; yellow star: G3) **(B)** and nuclear distribution **(C)**.

Because of their high occurrence, these mutations have been studied extensively using *in vitro* structural, *in situ* cell culture and *in vivo* mouse model approaches (Tillotson and Bird, 2019). Within the first category, we have already referred to the early pioneering work on R106W, R133C, F155S, and T158M by Ballestar et al. (2000). This structural work was followed by a study of R106W, R111G, R133C, F155S, and T158M, which, in the absence of crystallographic MBD information (Ho et al., 2008), used NMR and provided a more detailed molecular characterization of the structural changes resulting from these mutants (Free et al., 2001). It was noticed that the R133C mutation affected DNA binding without changing the MBD structure, thus highlighting, for the first time, the relevance for proper DNA binding of the basic amino acids at the MBD-DNA interface (Spiga et al., 2019). These studies were ensued by a later characterization of the same mutants using a combination of biophysical techniques that included fluorescence spectroscopy and circular dichroism. They allowed the authors to correlate the magnitude of the structural changes elicited by each mutant to the severity of the associated RTT phenotypes (Ghosh et al., 2008). Due to the complexity of the structural work, initial studies focused on some of the most prevalent RTT mutations and only more recently have been extended to other mutants such as the Y120D and to their binding to mCH (where H = A, T, or C) (Sperlazza et al., 2017), and in particular to mCA (Gabel et al., 2015). Although no structural differences were observed between the binding of the MeCP2 mutants to mCG versus mCA (Sperlazza et al., 2017), this study was very relevant as, immediately after birth, during neuron differentiation, mCH (Lister et al., 2013) and MeCP2 (Kaufmann et al., 2005b; Olson et al., 2014) concomitantly increase during a changing methylation landscape that may account for the onset of RTT (Lavery and Zoghbi, 2019). From the structural point of view, the different missense MBD-RTT associated mutations can change either the stability of the MBD, its DNA binding affinity, or both to a different extent. In this regard, a couple of recent exhaustive structural studies summarize this in a way that clusters these mutations into three different groups (Kucukkal et al., 2015; Yang et al., 2016) (see Table 1 and Figure 3).

**TABLE 1:** Classification of MeCP2 MBD missense mutations according to their structural characteristics (Yang et al., 2016).

**Group (Cluster)**	**Mutation (No. of cases)** (Krishnaraj et al., 2017; Spiga et al., 2019)	**Structural characteristics**
Group 1	L100V (7) S134C (21) P152R (71) D156E (15)	MBD propensity to unfold and reduced binding affinity for C methylated DNA
Group 2	R106W (132) R106Q (21) R133H (8) R133C (217) F155S (2) T158M (419) T158A (2)	No major changes in MBD structure but various binding affinities for C methylated and unmethylated DNA
Group 3	R111G (1) A140V (29)	Loss of MBD flexibility leads to reduced binding affinity for either C methylated or unmethylated DNA

The information from the structural studies has been complemented by *in situ* experiments in different cell culture settings. A few representative papers covering a wide spectrum of mutations have been published by Kudo et al. (2003), and by Agarwal et al. (2007). In addition to stability and affinity of binding, these studies have also focused on the clustering ability of MeCP2 around the pericentromeric heterochromatin. Importantly, the work has also provided insight to the altered distribution within the nuclear/cytoplasmic compartments caused by the MeCP2 mutations (Figure 3C). As with the *in vitro* structural work, an attempt has been made to correlate the observations made with clinical severity (Sheikh et al., 2016; Figure 3C). Interestingly, while some of the MeCP2 protein mutants resulting from MBD mutations (i.e., R133C and A140V) are still able to bind to chromatin, their interaction with ATRX is fully compromised (Nan et al., 2007). ATRX is a protein member of the ATP-dependent SWI/SNF family of chromatin remodeling complexes (Pazin and Kadonaga, 1997). This additional disruption of a functionally relevant protein-protein interaction underscores the molecular mechanistic complexity of some of these mutations.

In more recent years, a few knock-in mice models have been produced for the Y120D, R133C and T158M/A mutations as well as two transgenic models for the R111G and R306C mutations (Heckman et al., 2014). These models have provided useful information from an *in vivo* perspective. This work has revealed that, in instances such as T158M, where the mutation significantly decreases the amount of MeCP2 in the nucleus, the RTT phenotype can be rescued by increasing the expression of the T158M mutant (Lamonica et al., 2017). Moreover, the decrease in the mutated MeCP2 was shown to be due to proteasomal degradation (Lamonica et al., 2017). This decrease is reminiscent of that observed for the truncated form of MeCP2 expressed in the Jaenisch (*Mecp2 ^TM 1^.^1*Jae*^/*Mmcd) mouse model (Stuss et al., 2013), in which exon 3 of MeCP2 is deleted (Figure 1A) such that most the MBD is lacking (Chen et al., 2001).

#### MeCP2 Nonsense and C-Terminal Mutations

In this section, we include all the mutants affecting MeCP2 beyond its MBD. These include mutations affecting the ID, TRD, and CTD domains that encompass the NID and RNA binding domain (RBD) (Figure 3A). The C-terminal region defined in this way, is also where MeCP2 mutations pertaining to other brain disorders such as schizophrenia, which involves the ID, (Sheikh et al., 2018; Chen et al., 2020) take place. Many of the RTT nonsense mutations occur within this region and its most significant missense mutations take place in the TRD (Figure 3A). From a structural perspective, mutations within this region have been less extensively studied, in contrast to those of the MBD. This is particularly true as it pertains to the nonsense and frameshift mutations leading to early termination. In this regard, a hint into some of their potential molecular effects can be envisaged from the early structural work carried out in the late Alan Wolffe’s lab (Chandler et al., 1999; Yusufzai and Wolffe, 2000) which used C-terminally truncated versions of MeCP2 to show that the CTD facilitates its binding to nucleosomes (Chandler et al., 1999) and hence to chromatin. A more detailed follow up, carried out by Nikitina et al., highlighted the role of residues 295–486 for chromatin interaction (Nikitina et al., 2007b), and showed that R294X failed to produce the nucleosome–nucleosome interactions (Nikitina et al., 2007a) that are observed with the native form of the protein. Disruption of the inter-nucleosome interactions may play an important role in the intrinsic ability of MeCP2 to organize chromatin into chromocenters (Brero et al., 2005; Agarwal et al., 2011; Ausio, 2016; Wang et al., 2020). The RTT nonsense mutations, R168X, R255X, R270X, and R294X, which together account for about 90% of all nonsense mutations (Krishnaraj et al., 2017) were recently shown to disrupt the ability of MeCP2 to cluster heterochromatin (Li et al., 2020) in a way that progressively decreased with the proximity of the MeCP2 truncation to the C-terminus (Wang et al., 2020). These results agree with the clinical observations which indicate that severity of the C-terminal truncations decreases with its proximity to the carboxy-terminus of MeCP2, with individuals with the R168X mutation being more severely affected than those with R294X (a mild RTT mutation) or other more C-terminal mutations (Neul et al., 2008; Bebbington et al., 2010; Cuddapah et al., 2014). They also agree well with the recently described critical role of the unstructured MeCP2 CTD in conjunction with the MBD to form heterochromatin condensates (Li et al., 2020). Of note, the R255X and R270X mutations fall within the NLS region, however, the molecular relevance of this is not clear, especially since NLS inactivation does not affect the progression of the disease in a RTT mouse model (Lyst et al., 2018).

Given the confounding effects resulting from the mosaic expression of MeCP2 in females (XX), boys (XY) with RTT allow for a better correlation between mutation and phenotype severity. In this regard, boys with truncation or frameshift mutations before or including residue R270 exhibit neonatal encephalopathy and death, whereas males with the same type of mutations beyond G273 survive. Using R270X and G273X mouse models, the breaking point was shown to be due to the disruption of an AT-hook 2 HMGA1 (high mobility group)-like domain in MeCP2 that was found to be critical for chromatin maintenance and α-thalassemia mental retardation X-linked protein (ATRX) localization in the nervous system (Baker et al., 2013), underscoring again the multifaceted role of MeCP2 in chromatin organization. As with the chromatin architectural HMGA1 non-histone protein (Reeves, 2001), MeCP2 contains three homologous AT hook domains (Ausió et al., 2014) that provide the molecule with DNA binding properties involved in chromatin clustering and heterochromatin organization. Interestingly, HMGAs play an important role in the regulation of the neurogenic potential of neuron precursor cells, and their expression is lost during neuron differentiation (Tyssowski et al., 2014).

In addition to all the above, the possibility exists that the deleterious consequences of the nonsense mutations giving rise to the truncated forms of MeCP2 may also be partially indirect in nature. Indeed, analyses of the histone PTMs in lymphocytes from RTT patients showed a decrease in the levels of acetylation of lysines 9 and 14 of histone H3 (Kaufmann et al., 2005a). These analyses are interesting, and add to the promise of biomarker discovery in RTT patient lymphocytes, but unfortunately they do not provide insight into the potential molecular mechanisms involved. In experiments carried out on clonal cell cultures from an RTT female with the R168X mutant and cells from a male hemizygote for the frameshift mutation 803delG (V288X), both sets of mutant cells exhibited histone H4 hyperacetylation specifically associated with increased acetylation of lysine 16 (H4K16ac) (Wan et al., 2001). In a different study using a mouse model of Rett syndrome expressing MeCP2 truncated by introducing a stop codon after codon 308 (Mecp2^308/y^), a 2–3 fold increase in histone H3 acetylation was observed in cortex (Shahbazian M. et al., 2002). The changes in global acetylation observed in these studies might have important alterations in gene expression and in both instances had been attributed to the inability of these truncated versions of the expressed protein to recruit the MeCP2-associated histone deacetylase (HDAC) complexes (Nan et al., 1998; Jones et al., 2001). However, the relation of this acetylation to the MeCP2-dependent HDAC recruitment is surprising, as the null mouse model lacking the expression of MeCP2 does not exhibit any differences in histone H3 or H4 acetylation (Urdinguio et al., 2007). A more plausible explanation would be that MeCP2 might have a developmental-dependent downstream effect on gene expression (Thatcher and Lasalle, 2006) which is altered in different ways in the presence of different truncated MeCP2 forms and in different tissues.

The most important C-terminal missense mutations (in terms of frequency): P302R, K304E, K305R, and R306C occur in the TRD within the NID (Figure 3A). In this regard, R306C represents one of the most frequent mutations observed in RTT with 245 (5.1%) RTT cases reported (Krishnaraj et al., 2017). R306C suppresses MeCP2 binding to the nuclear receptor co-repressor (NCoR)-mediated recruitment of HDACs, which is also severely compromised by any of these four RTT mutations (Kruusvee et al., 2017). Yet, despite the functional relevance to RTT attributed to the MeCP2 NID (Tillotson et al., 2017), and despite the main functional role of MeCP2 in the brain being to recruit the NCoR1/2 co-repressor complex to methylated DNA sites in this tissue (Tillotson and Bird, 2019), mutations within this region correspond to some of the clinically milder RTT forms reported (Schanen et al., 2004; Cuddapah et al., 2014; Neul et al., 2014). Also, the genomic sites to which the NCoR1/2 complex is recruited (Connolly and Zhou, 2019) and their relative abundance are still unknown.

Other molecular implications for the mutations within the MeCP2 C-terminus could arise from the fact that the CTD had been shown early on to contain a WW domain binding region (WDR) encompassing amino acids 325–498. This region is responsible for the interaction of MeCP2 with group II WW-containing domains in splicing factors FBP11 and HYPC (Buschdorf and Stratling, 2004). Several missense mutations and small C-terminal INDELs causing truncations occur within this region (Kyle et al., 2018; Lavery and Zoghbi, 2019; Spiga et al., 2019), the most prominent being E395K (Krishnaraj et al., 2017). However, structural information for any of these is still lacking.

#### Proteasomal Degradation and the PEST Sequences

As has already been mentioned above for various MeCP2 mutations, several of the anomalous cellular levels and pathological aspects involved arise from alterations in the proteasomal degradation processing of these mutants (Lamonica et al., 2017; Sheikh et al., 2017). Whether the proteasome activity takes place in the nucleus or in the cytoplasm is not yet clear and it may be dependent on the type of mutation. While both the N terminus-dependent pathway and the lysine-dependent (PEST) degradation pathways can occur in the nucleus, the latter occurs more actively in the cytoplasm (Lingbeck et al., 2003). Hence, the cellular localization of the MeCP2 protein molecules to be degraded might be mutation dependent. Indeed, the MBD by itself is important for nuclear localization (Lyst et al., 2018) and hence MeCP2 partitioning within the cell might be affected by impairment of its ability to bind to its methylated DNA target.

Regardless of the cellular compartment where the degradation of MeCP2 mutants takes place, the ubiquitin proteasome system (UPS) plays a very important role in neurological diseases, including cognitive disorders like RTT (Lehman, 2009). Mesenchymal stromal cells from a heterozygous RTT female mouse model null for MeCP2 (Mecp2tm1.1Bird), which mimics partial MeCP2 loss of function, have been shown to exhibit increased proteasome activity (Squillaro et al., 2019). Similar observations associated with changes in cellular ubiquitination have been described for peripheral blood lymphomonocytes from RTT patients (Pecorelli et al., 2013).

In view of all of this, we propose here a mechanism for MeCP2 degradation of missense mutations that relies on the presence of two PEST domains in MeCP2 (Thambirajah et al., 2009; Figure 3B). These domains consist of consensus sequences enriched in proline, glutamate, serine, and threonine (PEST) residues, which act as a recognition signal for rapid degradation by the 26S UPS (Rogers et al., 1986; Rechsteiner and Rogers, 1996). The two PEST domains of MeCP2 are N-terminally and C-terminally located at amino acid residues 73–94 and 389–426, respectively (Thambirajah et al., 2009). However, whether this mechanism would apply to all the RTT mutations it is not clear and, as in the case of the N-terminal mutations, additional mechanisms may also apply (Sheikh et al., 2017). While limited detailed information is available for some of the N-terminal (Sheikh et al., 2017) and MBD missense (Lamonica et al., 2017) mutations, information in this regard on mutations occurring at other MeCP2 domain locations is significantly lacking, and information on stability and binding affinity it is only known in a few instances. Such is the case of the reduced DNA binding affinity of the R306C mutation described in the previous section, which affects the TRD/NID (Figure 3A). Using a mouse model for this mutation, Heckman et al. have conclusively shown that, beyond the impairment of binding the repressive NCoR complex (Kruusvee et al., 2017), alteration of the basic cluster of basic amino acids (304–309) within the RNA binding domain (Figure 3A) by R306C lowers the binding affinity of the mutant protein by MeCP2 binding sequences *in vivo* (Heckman et al., 2014). Importantly, it might also disrupt the interaction of the protein with RNA as will be described next.

### MeCP2 as an RNA Interacting Protein

#### MeCP2 RNA Binding Domain(s)

Direct interaction between MeCP2 and RNA was first shown by *in vitro* electrophoretic mobility assays (Jeffery and Nakielny, 2004). MeCP2 shifted mouse immunoglobulin mRNA and *Xenopus* U1 spliceosomal small nuclear (sn)RNA, but not human tRNA or *Xenopus* 5 S rRNA, suggesting that RNA binding is not promiscuous. Removal of an RG repeat motif C-terminal to the MBD abolished RNA binding (Figure 2A). Of note, double stranded (ds), but not single stranded (ss) RNA was shown to compete with methylated DNA, implying mutually exclusive binding to methylated DNA or dsRNA. Despite these promising but preliminary results, very little further research has been performed to characterize MeCP2-RNA interactions. It is becoming increasingly clear that protein interactions with both DNA and RNA are key to almost all nuclear processes, particularly because of the emerging regulatory roles played by lncRNAs (Hudson and Ortlund, 2014). RG repeat motifs are well established RNA binding modules (Thandapani et al., 2013). The binding preference for these motifs is debated, but evidence tends toward affinity for G-quadruplexes or GC-rich dsRNA, where arginine residues form hydrogen bonds with guanines (Jarvelin et al., 2016). This allows for almost transcriptome-wide binding possibilities, where specificity might be modulated by structural context and/or residues commonly occurring near RG repeats, such as the PGG, GGG, and polyalanine residues found in MeCP2 (Figure 2A; Chong et al., 2018). MeCP2 has a relatively short RG repeat, which is generally associated with low RNA binding affinity. An AT-hook domain overlaps the RG repeat, which was later characterized as a non-canonical extended AT-hook (eAT-hook) (Figure 2A; Filarsky et al., 2015). eAT-hook proteins have an order of magnitude greater affinity for dsRNA than DNA, and long stem-loop RNA structures are preferred over short hairpins (Figure 2C).

Advanced proteome-wide screens of RNA binding proteins (RBPs) have identified MeCP2 as an important RBP (He et al., 2016; Trendel et al., 2019). Recent comprehensive *in vivo* capture of RBPs identified a lysine-rich non-canonical RNA binding motif within the TRD of MeCP2 (Castello et al., 2016). RNA binding domains (RBDs) that lack sequence homology to known RBDs are being found with increasing frequency, and are presently characterized by basic (R and K) and disorder-promoting (R, G, P, S, and Q in MeCP2) residues. K-rich regions in DNA binding domains are thought to allow “hopping” or “sliding” to specific sequences; however, K-rich RNA binding domains characterized to date imply RNA structure over sequence-specific binding (Wilson et al., 1998; Takeuchi et al., 2009; Castello et al., 2016; Figure 2C). Most non-canonical RBDs are also enriched in having DNA and protein interaction surfaces, suggesting competition between RNA binding and other molecular interactions at these regions. This agrees with the K-rich RBD of MeCP2 overlapping its NID, a region within which RTT-causing missense mutations are also enriched (Figure 2A). RTT-like phenotype rescue in knock-in *Mecp2*-null mice expressing a minimal MeCP2 protein lacking the N- and C-terminal regions as well as the intervening region suggest that just the MBD and NID are necessary and sufficient for MeCP2 function (Figure 2A; Tillotson et al., 2017). The CTD probably plays a minor functional role, as its absence caused mild intellectual phenotypes. The discrepancy between a minimal peptide and diverse functionality of the protein has been reconciled by concluding that the dominant role of MeCP2 is to mediate transcriptional repression via NCoR complex recruitment to methylated DNA. An alternative explanation could be that the regions containing the MBD and/or NID mediate some degree of protein multifunctionality (Ghosh et al., 2010). Surface plasmon resonance assays show that MeCP2-TBLR1 (the direct binding subunit of the NCoR complex) interaction is relatively weak (K_D_ 9.5 ± 0.5 μM), which could be due to a lack of physiological context *in vitro*, or it could indicate transient binding (Kruusvee et al., 2017). Evidence for NCoR mutations specifically causing Rett syndrome is lacking (Sakaguchi et al., 2018; Zaghlula et al., 2018). Instead, they can cause intellectual disabilities, rather than RTT, *per se*, similar to misregulation of other MeCP2-associated processes such as mRNA splicing or miRNA biogenesis, suggesting comparable importance of MeCP2’s differing roles (Young et al., 2005; Ha and Kim, 2014). Given a growing catalog of diverse regulatory RNAs, the presence of a flexible RBD within the indispensable NID region of MeCP2 is a good candidate to explain its functional multiplicity. Moreover, combinatorial action between the K-rich RBD and the eAT-hook/RG repeat region of MeCP2 could add to the complexity.

Post-translational modifications (PTMs) are another regulatory mechanism of RBPs, some of which MeCP2 may share (Xu et al., 2019). Conservation analysis of PTMs found within RBDs showed that phosphoserine is often immediately preceded by a conserved glycine, and phosphothreonine can often be found 5 amino acids upstream from a conserved serine such as that seen on MeCP2 residues S274 and T308 (Figure 2A; Castello et al., 2016). Both PTMs have been experimentally determined (Bellini et al., 2014). Differential activity-dependent mechanisms determine phosphorylation of S274 (protein kinase A) and T308 (membrane depolarization), where phosphorylated T308 abrogates NCoR binding, potentially also representing differential activity-dependent mechanisms of RNA binding regulation (Ebert et al., 2013). MeCP2 is also ubiquitinated (K271) and acetylated (K271, K289, K305 or 307) at sites within the RBD (Gonzales et al., 2012; Pandey et al., 2015). The roles of these PTMs are unclear, but ubiquitination can influence protein conformation, and acetylated K305 could be important for protein function, as indicated by the acetyl-defective RTT mutations, K305E/R (Ausió et al., 2014). Methylation at R162 within the RG repeat motif could affect affinity for RNA, as is common for other RGG/RG proteins (Blackwell and Ceman, 2012; Guo et al., 2014). PTMs outside of RBDs can also modulate RBP function, allowing for further positive or negative regulation of RNA processing and fate (Lovci et al., 2016). This may also be true for MeCP2, similar to how activity-dependent phosphorylation outside the MBD bi-directionally regulates DNA binding (Tillotson and Bird, 2019). Altogether, the available data support MeCP2-RNA binding, but the role(s) RNA may play, and how the K-rich and/or RG repeat motifs are involved, can only be speculated as of now.

#### mRNA Splicing Regulation

Methyl CpG binding protein 2 has been shown to increase exon inclusion of a CD44 minigene reporter through RNA-dependent interaction with the YB-1 splice factor in HeLa and Neuro2A cells (Young et al., 2005). MeCP2 pulls-down YB-1 through TRD residues, but a C-terminal RTT-causing truncation, MeCP2-308X, binds less efficiently to YB-1, reducing exon inclusion. MeCP2 also immunoprecipitates CD44 precursor (pre-) mRNA, and MeCP2-308X abrogates this binding. Given that MeCP2’s putative RBDs do not overlap with the CTD (see above), the pre-mRNA interaction may be indirect, or direct pre-mRNA binding could require CTD-protein binding, PTMs, or structural context. Wild type and *Mecp2^308/Y^* mice have significantly altered genome-wide alternative splicing (AS), including that of *Dlx5* and *Cdk10* – direct targets of MeCP2-mediated repression and activation, respectively. Activity-dependent dephosphorylation at Serine pS80 enhances MeCP2-YB-1 interaction, suggesting MeCP2-dependent splicing regulation occurs in the brain (Gonzales et al., 2012).

In addition to YB-1, MeCP2 binds many splicing factors in different contexts, primarily through the CTD, and to a lesser extent the transcriptional repression domain (TRD) (Table 2; Buschdorf and Stratling, 2004; Long et al., 2011; Maxwell et al., 2013). The RNA-binding or RTT relevance of these interactions also vary, or are unknown. MeCP2 assembles with pre-mRNA processing factor 3 (Prpf3) and serologically defined colon cancer antigen gene 1 (Sdccag1) to pre- and mature mRNA of the MeCP2 gene targets *Cdk10* and *Frg1* (Long et al., 2011). The number of documented MeCP2 splice factor interactions continues to grow, with one article even reporting that the majority of MeCP2-bound proteins are involved in RNA splicing and processing (Cheng et al., 2017). The spliceosome is a massive macromolecular complex with many auxiliary proteins providing context-specific AS, so it is unsurprising that such variation exists and that concrete ties to RTT pathology have been difficult to make.

**TABLE 2:** MeCP2 interacting splicing factors.

**Splicing Factor**	**Tissue/Cell type**	**Methods**	**MeCP2 interaction site**	**RTT mutations abrogate binding?**	**RNA dependent?**	**Ref**
FBP11, HYPC	HEK293	GST pull-down and Co-IP	From 325 (CTD)	Yes, C-terminal truncations	Untested Untested	Buschdorf and Stratling, 2004
YB-1	HeLa, Neuro2a	GST pull-down and Co-IP	195–329 (TRD)	Yes, C-terminal truncations	Yes	Young et al., 2005
Prpf3 Sdccag1	Whole rat brain nuclei	GST pull-down and Co-IP	104–141 (MBD), 207–294 (TRD) From 311 (CTD)	Yes, C-terminal truncations	No No	Long et al., 2011
Prp8 Top2b DXH9	Whole mouse brain nuclei	Co-IP, MS	Unmapped	Untested	No Yes Yes	Maxwell et al., 2013
LEDGF, DXH9	*MeCP2-Flag* KI whole mouse brain nuclei	Co-IP	163–270 (TRD) Unmapped	Yes, TRD truncations Untested	No No	Li et al., 2016
TDP-43, FUS, hnRNP F					No No No	

C-terminal truncations account for ∼10% of RTT cases, yet significant functional relevance has yet to be attributed to this domain. Several truncations tested in the articles above coincide with known Rett syndrome genotypes, suggesting correlation with splice factor binding.

Most reported MeCP2-mediated AS events are cassette exon inclusion and intron exclusion (Young et al., 2005; Wong et al., 2017; Osenberg et al., 2018). Intron exclusion events were aberrant for MeCP2 target gene transcripts, *Dlx5* and *Cdk10*, in *Mecp2^308/Y^* mice (Young et al., 2005). RNA-seq analysis in *Mecp2* KO mouse cortex, however, suggests bidirectional roles in several types of AS (Li et al., 2016). In addition to protein-RNA-mediated regulation, AS is intimately tied with DNA methylation, and MeCP2 binds methylated exonic DNA, stalling RNA Polymerase II (RNAPII), thus reducing the chance of skipping alternative exons (Maunakea et al., 2013). These initial findings did not distinguish methylation (5mC) from hydroxymethylation (5hmC). However, more discriminatory experiments reveal enrichment of 5hmC at exon-intron boundaries in neurons, whereas 5mC exon-intron enrichment is prevalent in non-neuronal cells, supporting a role for 5hmC in MeCP2-mediated AS in the brain (Khare et al., 2012; Wen et al., 2014; Li et al., 2016). This agrees with MeCP2 enrichment on exon-intron gene boundaries and on 5hmC at active neuronal genes during postnatal development (Kinde et al., 2015; Li et al., 2016).

Alternative splicing is a highly conserved process allowing for greater protein and ncRNA diversity than provided by individual genes (Weyn-Vanhentenryck et al., 2018). A plethora of splice factors with different expression profiles is essential for correct AS during development. Cassette exon inclusion increases in mouse brain during development, which correlates with the increase in MeCP2 expression (Olson et al., 2014; Weyn-Vanhentenryck et al., 2018). MeCP2 interaction with the spliceosome and with pre-mRNA occur primarily through the CTD, suggesting some RTT pathologies from C-terminal truncations could, to some extent, derive from aberrant AS. Of note, during the writing of this manuscript, two research articles related to MeCP2’s role in AS were published. In the first, quantitative assessment of high-quality sequencing datasets found little variation in global AS as a result of differential MeCP2 and/or DNA methylation levels (Chhatbar et al., 2020). However, in the second article, MeCP2 was found to be required for maintaining mature hippocampal AS profiles, and to regulate splicing of specific neuronal genes in the hippocampus during memory consolidation (Brito et al., 2020). These two recent papers underscore the still long road ahead in understanding MeCP2’s role in AS, as well as the importance of careful context-specific interpretation of MeCP2 studies moving forward.

#### miRNA Biogenesis and Binding

MicroRNAs (miRNAs) represent an important class of ∼22 nucleotide molecules with key roles in regulating the translation of the proteome (Ha and Kim, 2014). Genome-wide miRNA expression levels are aberrant in the brains of Rett syndrome patients, and offer a potential tool to measure RTT disease progression and treatment response (Wu et al., 2010; Sheinerman et al., 2019). Processing primary (pri-) miRNA into precursor (pre-) miRNA by the nuclear microprocessor complex before export to the cytoplasm is the key regulatory step in determining mature miRNA levels in the cell (Conrad et al., 2014). The core microprocessor proteins are Drosha, which cleaves the pri-miRNA, and DiGeorge syndrome critical region 8 (DGCR8), which provides RNA binding affinity to Drosha (Ha and Kim, 2014). Additional development and cell-type specific co-factors regulate microprocessor activity. In addition to MeCP2, a major atypical RTT-pathogenic protein, FOXG1, is recruited to Drosha to influence miRNA biogenesis, implicating the importance of this process to RTT pathology (Weise et al., 2019).

At a resting state, phosphorylated MeCP2 pS80 inhibits miRNA biogenesis in cultured rat cortical neurons by binding and sequestering DGCR8; activity-dependent dephosphorylation reduces this interaction, allowing miRNA processing to proceed (Cheng et al., 2014). In *Mecp2-*KO mice, miR-134 increases, resulting in decreased levels of its targets involved in neuronal development and plasticity, in addition to reduced dendritic growth. Another group corroborated MeCP2 regulation of miRNA processing through microprocessor interaction, but with some key differences that require reconciliation (Tsujimura et al., 2015). Here, MeCP2 was found to positively regulate miRNA levels in neurons and neural stem cells (NSCs). The authors posited that the different state of MeCP2 phosphorylation, which varies between neuron types and brain regions, could explain the seemingly opposite observations. A global screen of significantly reduced miRNAs in *Mecp2*-KO neurons and NSCs identified miR-199a as important to RTT pathophysiology due to its positive regulation of mechanistic target of rapamycin (mTOR) signaling. MeCP2-mediated miR-199a biogenesis results in targeted inhibition of mTOR inhibitors SIRT1, HIF1a, and PDE4D. SIRT1 deacetylates MeCP2, adding the possibility of feedback regulation by acetylation level, in addition to phosphorylation (Zocchi and Sassone-Corsi, 2013). Furthermore, phosphorylation of DGCR8 by the mTOR-kinase ERK increases its stability (Herbert et al., 2013). RNA immunoprecipitation (RIP) shows either direct or indirect *in vivo* interaction of MeCP2 with pri-miR199a-1 and pri-miR199a-2. Similar to a proposed mechanism of alternative splicing (see above), MeCP2 binds to methylated miRNA gene boundaries, stalling RNAPII, and enhancing miRNA biogenesis by permitting access to processing machinery (Glaich et al., 2019). It could be that methylation level and MeCP2-DNA binding promote miRNA biogenesis, such as with miR199a, whereas unmethylated miRNA genes are subject to alternative MeCP2-mediated miRNA biogenesis suppression, like in Cheng et al. (2014). The above data offer tenuous support of direct MeCP2-RNA binding in miRNA processing regulation. It is intriguing to speculate a correlation between MeCP2 interaction with paraspeckle lncRNA, NEAT1, which can scaffold Drosha and DGCR8 to peripheral paraspeckle proteins, resulting in the regulation of miRNA biogenesis (Jiang et al., 2017). MeCP2 is known to bind the long isoform of NEAT1 in the brain (see below) (Cheng et al., 2018).

In addition to pri-miRNAs, RIP identifies 87 mature nuclear miRNAs associated with MeCP2 in primary mouse cortical cells (Khan et al., 2017). All MeCP2-interacting miRNA target gene sets are inhibited in *Mecp2*-null mouse cerebellum, implying an inhibitory role of MeCP2 on mature miRNAs, thus positively targeting gene expression. In addition to the canonical role of miRNAs in mRNA decay, nuclear miRNAs are associated with transcriptional repression and activation, as well as alternative splicing (Roberts, 2014).

miRNAs are essential to mammalian cell function, and their aberrant regulation as a result of MeCP2 mutations likely contributes to RTT phenotypes, but the exact interplay of molecular interactions, and whether RNA is directly involved, is complex and remains unclear.

#### lncRNA Interactions

Long non-coding RNAs (lncRNAs) are >200 base molecules with low coding potential, the varying species of which are involved at every processing step in the nucleus (Zhang et al., 2019). Tissue-specific expression patterns of lncRNAs during development are highly dynamic, allowing diverse outcomes, and are thus unsurprisingly aberrant in RTT (Petazzi et al., 2013; Hosseini et al., 2019). Protein-lncRNA interactions occur with all major classes of epigenetic modifying complexes (Betancur, 2016). Notably, all known RNA-binding subunits of lncRNA-interacting epigenetic complexes lack a canonical RNA binding region, and have at least some level of disorder, similar to MeCP2. Currently, there are four lncRNAs whose interaction with MeCP2 has been reported: *Evf2*, *RNCR3*, *Neat1L*, and *HSATII*, as discussed below.

During embryonic GABAergic neuron development, *Evf2* recruits MeCP2 and the transcriptional activator distal-less homeobox 1 (DLX1) to the *Dlx5/6* homeotic gene cluster, and inhibits DNA methylation there to facilitate antagonism between the two proteins (Berghoff et al., 2013). *Evf2* deletion leads to impaired synaptic connectivity, and *Mecp2*-KO as well as common RTT mutations present GABAergic defects (Horike et al., 2005; Schule et al., 2007). *Evf2* also recruits the SWI/SNF-like chromatin remodeling complex protein brahma-related gene 1 (BRG1) to *Dlx5/6*, which has overlapping protein and RNA binding motifs, similar to MeCP2 (Cajigas et al., 2015). These data led to an important speculation that global DNA binding proteins that paradoxically cause specific intellectual phenotypes when dysregulated, such as MeCP2 in RTT or BRG1 in Coffin-Siris syndrome, may be regulated by specific lncRNAs like *Evf2*. RNA immunoprecipitation of mouse cerebellum robustly pulled down the retinal non-coding RNA (*Rncr3*), followed by metastasis associated lung adenocarcinoma transcript 1 (*Malat1*) (Maxwell et al., 2013). *Rncr3* is upregulated during retinal development, whereas its expression is reduced in *Mecp2*-null mice (Blackshaw et al., 2004). *Rncr3*^–/–^ mice display hindlimb clasping, small brain size, and aberrant axonal sprouting, similar to RTT phenotypes, but these phenotypes are attributed to miR-124a, which is expressed from the *Rncr3* gene (Sanuki et al., 2011).

Aberrant expression of pericentric *HSATII* RNA occurs in several cancers, and recruits polycomb group complex 1 (PRC1) as well as MeCP2 and its protein partner Sin3a into large nuclear condensates (Landers et al., 2020). These epigenetic factors are sequestered from regular function, facilitating genomic instability and cancer development. *HSATII* RNA is also aberrantly enriched in Parkinson’s disease patient blood samples, and MeCP2 regulates pericentric heterochromatin regions in neurons in an RNA-dependent manner, suggesting a role for *HSATII* RNA with MeCP2 in intellectual disorders (Billingsley et al., 2019; Marano et al., 2019). A paper published during the preparation of this manuscript showed that MeCP2 and major satellite RNA cooperate to organize pericentric heterochromatin, and that RNA interaction depends on the TRD (Fioriniello et al., 2020). This is consistent with the presence of an RBD overlapping MeCP2’s NID, as mentioned earlier.

Huntington’s disease (HD) studies found that MeCP2 binds and inhibits the long isoform of nuclear enriched abundant transcript 1 (*Neat1L*) lncRNA in various neuronal and brain tissue types (Cheng et al., 2018). MeCP2 inhibits *NEAT1L* through RNA rather than DNA interaction in wild type cells, whereas MeCP2 is reduced in HD, and increased *NEAT1L* levels protect against the mutant HTT gene. *NEAT1L* increases expression of anti-inflammatory and growth factors including peroxisome proliferator activated receptor-γ (PPARG), Nuclear Factor Kappa B Subunit 1 (NFκB1), and brain-derived neurotrophic factor (BDNF), which are also targets of MeCP2-mediated repression (Martinowich et al., 2003; Mann et al., 2010; Kishi et al., 2016). The short (3,735 nt, *Neat1S*) and long (22,741 nt, *Neat1L*) NEAT1 transcripts are ubiquitously expressed in mammalian cells, and the long isoform is required for the formation of massive ribonucleoprotein paraspeckles, which are heavily implicated in transcriptional regulation (Yamazaki et al., 2020). *Neat1*, *Malat1*, and *Evf2* are upregulated upon neuronal differentiation, similar to MeCP2 (Olson et al., 2014; Roberts et al., 2014). Overall, the data point toward important corollary roles for lncRNAs and MeCP2 during brain development. Further research of their interactions with MeCP2 are integral to understanding how they may relate to RTT. Of note, RNA has been shown to promote the formation of spatial compartments in the nucleus (Quinodoz et al., 2020), and might assist MeCP2 in its formation of heterochromatin condensates (Sheikh et al., 2016; Li et al., 2020).

#### Histone PTMs and lncRNAs

Given that MeCP2 repression occurs primarily through HDAC recruitment, it would be expected that MeCP2 deletion invariably increases histone acetylation levels. However, as we discussed earlier (section “MeCP2 Non-sense and C-Terminal Mutations”), studies to date have been conflicting (Wan et al., 2001; Balmer et al., 2002; Kaufmann et al., 2005a; Thatcher and Lasalle, 2006; Lilja et al., 2013). As explored in the previous section, lncRNAs are a promising means to explain disparate functions of the same protein complexes. Proteome-wide analysis of RNA-dependent protein complex formation shows that the Sin3a complex, including HDACs 1 and 2, requires RNA, whereas the NCoR1 complex, including HDAC3, forms independent of RNA (Caudron-Herger et al., 2019). This is consistent with mutually exclusive MeCP2 binding to RNA or NCoR, as suggested by their overlapping binding domains (Figure 1). Moreover, Sin3a has been shown to bind lncRNAs in the brain (Dharap et al., 2013). Whether there is any specific binding and regulation of MeCP2 together with Sin3a and its associated HDACs by lncRNA, however, has yet to be determined.

In addition to methylated DNA, MeCP2 has been shown to interact with histone methylation marks associated with constitutive and facultative heterochromatin: di-methylated histone H3 lysine 9 (H3K9me2) and tri-methylated histone H3 lysine 27 (H3K27me3), respectively, in mouse brain nuclear extracts (Thambirajah et al., 2012). A recent report shows that MeCP2 preferentially binds nucleosomes with H3K27me3 via the MBD (Lee et al., 2020; Figure 2A). Also, the genomic distribution of the DNA and histone methylation marks overlap, and MeCP2 differentially regulates transcription depending on H3K27me3 and H3K9ac profiles. MeCP2 also associates with histone methyltransferases G9a, protein arginine methyltransferase 6 (PRMT6), and euchromatic histone-lysine N-methyltransferase 1 (EHMT-1) (Dhawan et al., 2011; Xue et al., 2013; Subbanna et al., 2014). MeCP2 increases H3K9 methylation in mouse fibroblasts (Fuks et al., 2003). The *Neat1* lncRNA interacts with EHMT-1 at select genes in neuronal cells, and *Neat1* knockdown decreases H3K9me2 and is associated with increased memory formation (Butler et al., 2019). Little is known about the relationship between MeCP2 and H3K27me3 in the context of RTT, except the previously mentioned findings in the brain, and contrasting findings by Zachariah et al. (2012) where MeCP2 overlaps more with constitutive than facultative heterochromatin marks in primary mouse cortical neurons. These contradictory findings may be explained by the different contexts, or the fact that H3K27me3 levels were compared to different normalizers. Both constitutive (H3K9me3 marked) and facultative (H3K27me3 marked) heterochromatin domains have been shown to be regulated or stabilized to some extent by lncRNA (Yang et al., 2015; Thakur et al., 2020). Despite convoluted results, MeCP2 has continued to be found associated with various histone PTMs and chromatin-modifying enzymes over the years. Whether they are relevant to altered gene expression profiles in RTT patients is still unclear, and will require scrupulous context-specific examination in the future to form conclusions. Potential regulation by previously unconsidered factors like lncRNAs adds to the complexity of the issue.

### Functional Roles of MeCP2 Beyond the Brain

Because of MeCP2 multi-functionality as well as its high abundance in the brain, alterations of MeCP2 have been involved in almost every single neurodevelopmental and neurodegenerative disorder of this organ (Ausio, 2016). Nevertheless, besides the brain, the protein is quite abundant in several other tissues, for instance in the lungs (Shahbazian M.D. et al., 2002), and the implications of MeCP2 mutations for RTT within this context represent one of the less studied areas in RTT research. It is thus highly possible that several symptoms observed in RTT do not simply arise from neurological disorders, but are also caused in part by disfunctional cellular regulation (Kyle et al., 2018) in organs other than the brain. Indeed, RTT patients often develop breathing issues and one of the most abundant causes of death in RTT is related to respiratory failure (Ehrhart et al., 2016).

In what follows, we will provide a few examples where MeCP2 has been shown to have an involvement that transcends the neural system and which might be of relevance to RTT.

MeCP2 plays an important role in the modulation of the immune system by influencing the expression of the transcription factor FOXP3 (fork head box P3) – a master regulator of T-helper and T-reg cells (Li et al., 2014). Thus, MeCP2 mutations can contribute to the pathogenesis of inflammatory disease in RTT. Moreover, intestinal isolates from RTT subjects show the presence of an altered microbiota and altered production of short chain fatty acids (Strati et al., 2016), and the presence of proinflammatory strains of *Candida parapsilosis* (Strati et al., 2018). The alteration in the microbiota may also contribute to the gastrointestinal pathophysiology such as constipation status (Motil et al., 2012; Strati et al., 2016). MeCP2 can also contribute to the development of rheumatoid arthritis (Miao et al., 2013).

Cardiac arrhythmia is one of the factors contributing to the greater than expected occurrence of sudden death in RTT individuals (Acampa and Guideri, 2006). Although the molecular involvement of MeCP2 is not completely understood, it appears that dysregulation of Rho GTPase cytoskeletal and inflammation mediated chemokine and cytokine signaling pathway genes are involved (Wang et al., 2018).

Osteopenia is another early symptom that RTT patients are at a risk of developing, and which is dependent on the MeCP2 mutation type (Caffarelli et al., 2020). Although the use of RTT murine models suggest an epigenetic regulation of bone (O’Connor et al., 2009) which might involve RANKL (receptor activator of nuclear factor-κB ligand) (Kitazawa and Kitazawa, 2007), which negatively regulates osteoblast differentiation and bone formation in bone marrow mesenchymal stem cells (Cao, 2018). Yet, the detailed molecular mechanism(s) by which MeCP2 is involved are not understood. This example underscores how the studies on the effects of MeCP2 mutations in non-neural cell types are still in their relative infancy, as this area of MeCP2 research remains understudied.

Metabolic dysfunction also represents an important component of RTT (Kyle et al., 2018). In this regard, it was recently shown that MeCP2 plays an important role in the regulation of liver homeostasis through a molecular mechanism that involves the targeting the NCoR1/HDAC3 complex to lipogenic gene targets in hepatocytes (Kyle et al., 2016). This underscores the relevance of the MeCP2/HDAC complex outside the neuronal realm. Moreover, lipid metabolism is a more approachable therapeutic target, offering the potential to alleviate the symptoms associated with altered metabolism in RTT patients (Kyle et al., 2016).

Although some of the RTT peripheral organ-related symptoms described above might also have a neuronal component (Cronk et al., 2016), the transcriptional regulatory role of MeCP2 of specific genes within the context of the cell types of the particular organs affected indicates an important role of the MeCP2 mutations within each specific tissue. As in the case of the impaired response to stimuli and stressors observed in RTT (Pillion et al., 2003; Rose et al., 2019), such specificity might also be MeCP2 isoform-dependent, as will be discussed in the next section.

### Do MeCP2-E1 and MeCP2-E2 Isoforms Play a Role in RTT?

**FIGURE 4:**
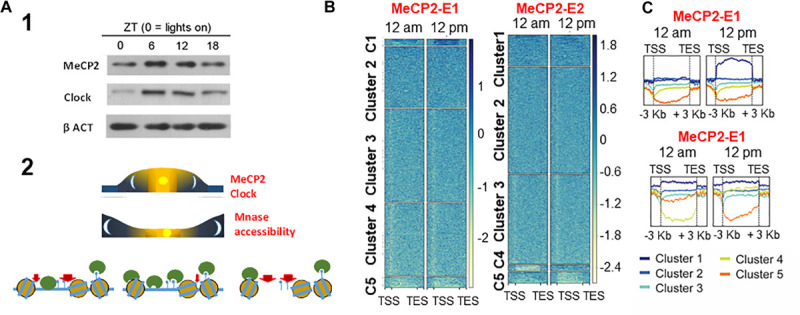
Circadian-dependent MeCP2 **(A-1)** and resulting chromatin changes **(A-2)** and diurnal dynamic change heatmaps for MeCP2-E1 and MecP2-E2 isoform gene occupancy **(B)**, divided into 5 clusters, with isoform-specific enrichment on the different clustered genes over time shown in **(C)**. MeCP2-E1 and MeCP2 enrichment differences in the different clusters in **(A)** (Martinez de Paz et al., 2015; Martínez de Paz et al., 2019).

Not only is the function of MeCP2 important in tissues other than the brain, but also within the brain its role transcends (Ausio, 2016, 2018) that of mere involvement in neurodevelopmental and neurodegenerative disorders (Tan and Zoghbi, 2019). For example, under healthy conditions, the levels of MeCP2 in mouse have been shown to change in a circadian cycle-dependent way (Martinez de Paz et al., 2015; Figure 4) – a mechanism which is likely regulated by miR-132/212 (Mendoza-Viveros et al., 2017) in response to the metabolism-dependent circadian cycle changes of the epigenome (Haws et al., 2020). This might have consequences for RTT (Tsuchiya et al., 2015). Indeed, a circadian rhythm disruption has been described in a mouse model of RTT, and disruption of the cycle was observed in fibroblasts from RTT patients (Li et al., 2015). RTT patients are known to frequently experience sleep disorders (McArthur and Budden, 1998). However, the implications of MeCP2 in the circadian cycle are undoubtedly much broader, and the system has allowed us to gain some insight into the different functionality of the E1 and E2 isoforms (Martínez de Paz et al., 2019).

Ironically, for several years most of the research on MeCP2 was carried out with the E2 isoform, which was the first to be identified (Lewis et al., 1992). However, it was not until almost 12 years later that a previously unknown MeCP2 isoform, which is much more highly expressed in human brain than its MeCP2-E2 counterpart, was discovered first in humans (Mnatzakanian et al., 2004), and then several months later in mice (Kriaucionis and Bird, 2004). The two isoforms are the product of alternative splicing. Despite an initially conflicting nomenclature, it was agreed that the longer E1 isoform corresponds to the encoded form starting at exon 1 (skipping exon 2) whereas the E2 isoform was the one encoded starting at exon 2 (Figure 1A). The physiological relevance of the two different isoforms of MeCP2 [MeCP2-E1 and MeCP2-E2 (Figure 1B)] and in particular, the relevance of the E2 form to RTT have been very controversial (Itoh et al., 2012). However, it is worth emphasizing that RTT mutations have never been identified within exon 2. In all these considerations, however, it is important to recognize that the ratio between the two isoforms and their overall abundance varies significantly from tissue to tissue (Mnatzakanian et al., 2004), and particularly in mature brain, MeCP2-E2 is present at a much lower ratio (approximately 15 fold less) than MeCP2-E1 (Martínez de Paz et al., 2019) as a result of their differential gene expression (Mnatzakanian et al., 2004).

In what follows, we will discuss the information available in support of a different specialized functional involvement of the two isoforms. As we mentioned in the previous sections, the occurrence so far of mutations in the amino acid distinctive MeCP2-E1 NTD suggests that only this isoform is relevant to RTT. However, as it was also mentioned earlier, mutations in exon 1 interfere with the translation of the MeCP2-E2 isoform (Saxena et al., 2006) and the possibility exists for other mutations along the MeCP2 protein to have a similar effect. At the gene level, the 5′ and 3′ UTRs of each isoform have been shown to be differently regulated in a cell type and development-dependent way (Liyanage et al., 2019; Rodrigues et al., 2020). At the 3′UTR, the transcripts undergo alternative polyadenylation affecting the length of these regions, ranging from 0.1 to 8.6 kb, with a preferential association of MeCP2-E1 with the longest 3′UTR (Rodrigues et al., 2020). Although how these differences regulate the expression of the isoforms is not clearly understood, they represent important targets for the binding of regulatory miRNAs and RBPs (Rodrigues et al., 2016). At the 5′ UTRs, it was reported that DNA methylation is significantly correlated with the differential expression of the two isoforms in neurons and astrocytes in a sex-dependent way, with higher levels of DNA methylation corresponding to lower levels of their expression (Liyanage et al., 2019).

As it was mentioned at the beginning of this section, recently, we took advantage of the circadian oscillation of MeCP2 to gain a functional insight on the role of the MeCP2-E1 and MeCP2-E2 isoforms. ChIP-seq analysis, taking advantage of the availability of isoform-specific antibodies, showed a differential binding site preference. MeCP2 isoform-specific enrichments were found to be mainly involved in ligand-receptor interaction in E1 and ribosomal proteins in E2 (Martínez de Paz et al., 2019). Of note, analysis of brains from RTT patients carrying MeCP2 mutations showed abnormal ribosome biogenesis (Olson et al., 2018).

At the protein level, a biophysical analysis using isothermic titration calorimetry and fluorescence spectroscopy analyses of the interaction of the E1 and E2 NTD-MBD protein region (Figure 1B) with methylated and unmethylated DNA showed a 10-fold higher affinity and higher structural stability of this region for the E2 isoform compared to the E1 counterpart. Half-life, MS, and FRAP (fluorescence recovery after photobleaching) analysis consistently reported a higher dynamic turnover of MeCP2-E1 compared to MeCP2-E2 (Sheikh et al., 2017; Martínez de Paz et al., 2019). Moreover, using isoform-specific antibodies, a proteomic analysis of the proteins interacting with the two isoforms revealed that, while both isoforms appear to be involved in similar processes, they act through different sets of protein partners. Of interest was the enriched association found between E1 and β-tubulin and microtubule-associated proteins (Martínez de Paz et al., 2019). The extent of overlap observed is, to a certain degree, unsurprising. It has been recently shown that MeCP2-E2 is able to partially compensate for the lack of the E1 isoform in a male case of RTT phenotype (Takeguchi et al., 2020). However, the association of E1 with tubulin remains intriguing. MeCP2 deficiency and mutations have been shown to affect microtubule stability (Delepine et al., 2013) and ciliogenesis (Frasca et al., 2020), respectively, through an indirect association between MeCP2 alteration and HDAC6 deacetylation of tubulin (Gold et al., 2015), though the molecular mechanisms are not yet clearly understood.

In conclusion, while the two MeCP2 isoforms may have a significant extent of generic overlapping functionality as a result of their extensive overlapping primary structure (Figure 1), they nevertheless exhibit important distinctive functional traits. Their effect(s) may depend on their different stoichiometry and overall abundance in different tissues as well as on the alteration of the mechanisms regulating their gene expression (Rodrigues et al., 2020), and their potential implications for RTT should not to be overlooked. Indeed, it has been recently shown that, in human brain, the MeCP2E1/E2-BDNF-*miR132* homeostasis regulatory network is region-dependent and is altered in RTT patients (Pehjan et al., 2020).

### Epigenetic Therapeutics

The area of therapeutics for RTT has become quite crowded over recent years. There are two main approaches, firstly that of addressing downstream effects of the MECP2 mutation, for instance attempting to upregulate genes under MeCP2’s control, such as BDNF or IGF1 [reviewed in Vashi and Justice (2019)], or neurotransmitter pathways such as NMDA receptors, or downstream target K^+^/Cl^–^ co-transporter 2 (KCC2) (Tang et al., 2019). The second approach is to target *MECP2* directly, either through gene therapy, delivering a functional version of the *MECP2* gene exogenously, for example using adenoviral delivery systems [e.g., (Tillotson et al., 2017)], through correcting the mutation at the level of genomic DNA, for example using CRISPR/cas9 editing, or at the RNA level by programmable RNA editing (e.g., Sinnamon et al., 2020), or using compounds such as aminoglycosides to enable “read-through” of MECP2 nonsense mutations [e.g., (Merritt et al., 2020)].

Attempts at epigenetic therapies for RTT, also within this second category of direct targeting of *MECP2*, would aim at the upregulation of *MECP2* expression. This in itself is a somewhat hazardous approach, as *MECP2* over-expression may also have severe developmental repercussions, as witnessed in *MECP2*-duplication syndrome (MIM # 300260). Since RTT is almost exclusively in females, who carry one normal copy of the *MECP2* gene along with a mutated copy, one approach that is being considered is reactivating the silent X [reviewed in Vashi and Justice (2019)]. Naturally occurring X-chromosomal inactivation (XCI) randomly silences one or other of the two X-chromosomes possessed by females epigenetically. This process allows dosage compensation of X-linked genes, which helps maintain the expression of most X-linked genes at a similar level to males.

Skewed XCI may favor the wild-type (WT) allele and hence expression of WT *MECP2*, in which cases RTT symptoms are milder, or, with extreme skewing, asymptomatic. If XCI skewing favors the mutant allele, expression of mutant *MECP2* is favored, and RTT symptoms would be more severe. XCI is an epigenetic process that occurs through the expression of an X-linked non-coding RNA, *Xist*. One potential therapeutic strategy for RTT (and other X-linked disorders) involves reactivating the inactive X chromosome in order to increase expression of WT *MECP2*, which should compensate for the loss of function (and/or expression) of the mutant *MECP2*. The drawback here is that X reactivation could potentially increase dosage of other X-linked genes to pathogenic levels, and so the challenge is to reactivate only *MECP2* or *MECP2* and its immediate genomic neighbors. Studies are still at an early stage, and there have been a number of high-throughput screens to identify molecules that can reactivate *MECP2* expression from the inactive X chromosome [e.g., (Minkovsky et al., 2015; Lessing et al., 2016; Sripathy et al., 2017)]. Subsequently, in one study, researchers used a small-molecule inhibitor of DNA methylation, 5-aza-2′-deoxycytidine, together with an antisense oligonucleotide knock-down of *Xist* RNA *in vitro*, that significantly upregulated *MECP2* expression, and *in vivo* using *Xist* knockout mice together with the 5-aza-2′-deoxycytidine-induced inhibition of DNA methylation successfully reactivated the inactive (Carrette et al., 2018a). In heterozygous *Mecp2* knockout mice with a mutation in *Tsix*, the antisense regulator of *Xist*, the phenotype observed resembled that of severely affected knockout null male mice, and demonstrated that small increases (5–10%) in WT MeCP2 protein expression can have dramatic improvements on the phenotype (Carrette et al., 2018b). The Tsix/Mecp2 mouse model generated in this study may prove to be an excellent preclinical model for evaluating the effects of XCI-based epigenetic therapeutic compounds.

## Author Contributions

KG, JV, and JA contributed equally to the preparation, writing, and revision of the manuscript. All authors contributed to the article and approved the submitted version.

## Conflict of Interest

The authors declare that the research was conducted in the absence of any commercial or financial relationships that could be construed as a potential conflict of interest.

## Footnotes

1 gnomad.broadinstitute.org

## Abbreviations

ATRX: α-thalassemia, mental retardation, X-linked protein
AS: alternative splicing
BDNF: brain-derived neurotrophic factor
BRG1: brahma-related gene 1
CD44: cluster of differentiation 44
Cdk10: cyclin-dependent kinase 10
ChIP: chromatin immune-precipitation
CTD: C-terminal domain
DGCR8: DiGeorge syndrome Critical Region 8
DNMT1: DNA methyltransferase 1
DLX1: distal-less homeobox 1
Dlx5: distal-less homeobox 5
eAT-hook: extended AT-hook
FBP11: formin-binding protein 11
FOXG1: fork head box G1
FOXP3: fork head box P3
FRG1: FSHD region gene 1
GABA: gamma aminobutyric acid
EHMT: euchromatic histone-lysine N-methyltransferase
ERK: extracellular signal-regulated kinase
EVF2: *Rattus norvegicus* non-coding RNA
HIF1a: hypoxia inducible factor 1 alpha
hmC: hydroxymethyl cytosine
HD: Huntington’s disease
HDAC: histone deacetylase
HMG: high mobility group
HSATII: human satellite II
HTT: huntingtin
HYPC: huntingtin yeast partner C
ID: intervening domain
IDP: intrinsically disordered protein
INDEL: insertions and deletion
lncRNA: long non-coding RNA
Malat1: metastasis-associated lung adenocarcinoma transcript 1
MBD: methyl biding domain
MeCP2: methyl CpG binding protein 2
mTOR: mechanistic target of rapamycin
NCoR: nuclear receptor co-repressor
ncRNA: non-coding RNA
NEAT1: nuclear enriched abundant transcript 1
NF κ B1: nuclear factor kappa B subunit 1
NID: NCoR interaction
NLS: nuclear localization sequence
NME: N-methionine excision
NTD: N-terminal domain
NSC: neural stem cell
PDE4D: cAMP-specific 3′-,5′-cyclic phosphodiesterase 4D
PEST: enriched in proline, glutamate, serine, threonine
PPARG: peroxisome proliferator activated receptor- γ
PRC1: polycomb group complex 1
PRMT6: protein arginine methyltransferase 6
PRPF3: pre-mRNA processing factor 3
PTM: post-translational modification
RANKL: receptor activator of nuclear factor-κB ligand
RBD: RNA binding domain
RBP: RNA binding protein
RIP: RNA immunoprecipitation
RNCR3: retinal non-coding RNA 3
RTT: Rett syndrome
Sin3A: switch independent 3 gene encoded protein a
SDCCAG1: serologically defined colon cancer antigen gene 1
SIRT1: Sirtuin 1
SMRT: silencing mediator of retinoic acid and thyroid hormone receptor
SWI/SNF: switch of the mating type/sucrose non-fermenting
TBLR1: transducin beta-Like 1X-Related protein 1
TRD: transcription repression
UPS: ubiquitin–proteasome system
UTR: untranslated region
WWDR: WW domain binding region
YB-1: Y box binding protein 1.
